# Multidisciplinary study of human remains from the 3rd century mass grave in the Roman city of Mursa, Croatia

**DOI:** 10.1371/journal.pone.0333440

**Published:** 2025-10-15

**Authors:** Mario Novak, Orhan Efe Yavuz, Mario Carić, Slavica Filipović, Cosimo Posth

**Affiliations:** 1 Centre for Applied Bioanthropology, Institute for Anthropological Research, Zagreb, Croatia; 2 Department of Archaeology and Heritage, Faculty of Humanities, University of Primorska, Koper, Slovenia; 3 Archaeo- and Palaeogenetics, Institute for Archaeological Sciences, Department of Geosciences, University of Tübingen, Tübingen, Germany; 4 Senckenberg Centre for Human Evolution and Palaeoenvironment at the University of Tübingen, Tübingen, Germany; 5 Archaeological Museum Osijek, Osijek, Croatia; 6 Department of Archaeogenetics, Max Planck Institute for Evolutionary Anthropology, Leipzig, Germany; University of Otago, NEW ZEALAND

## Abstract

During excavations in 2011, a peculiar archaeological feature representing a mass grave with seven completely preserved skeletons was discovered at the site of the Roman period city of *Mursa* (modern-day Osijek, Croatia). The archaeological context and direct radiocarbon dating indicate that the bodies were interred during the mid-3^rd^ century CE. Bioarchaeological analysis shows that all seven individuals are adult males exhibiting numerous pathological lesions (e.g., enthesopathies, injuries). Carbon and nitrogen stable isotopes analysis indicates they had a mixed C_3_/C_4_-based vegetal diet with limited amounts of terrestrial animal protein and a very limited marine protein consumption. Ancient DNA analysis shows that individuals from the *Mursa* mass grave had a heterogenous ancestry. None of them show genetic continuity with the preceding local Early Iron Age population. The presented multidisciplinary analyses of the *Mursa* mass grave strongly suggest that the studied individuals were Roman soldiers, victims of a catastrophic event occurring as the result of the ‘Crisis of the Third Century’, most probably the battle of *Mursa* from 260 CE.

## Introduction

Throughout its history, the Roman Empire produced every imaginable form of intentional violence, varying from individual violence such as homicide, through the community/societal scales of violence like gladiatorial games and sacrifices, to mass warfare. Today, numerous examples of Roman period violence (both archaeological and osteological) from Europe have been studied and published [1]. During the antiquity the remains of the victims of mass warfare such as battles and massacres were usually interred in mass graves without any special consideration for the deceased as can be seen on the examples from Skopje (*Scupi*) in North Macedonia [2], Hradisko in Czechia [3], and Ibida in Romania [4], just to name a few.

A comprehensive overview of documented Roman period mass burial sites was recently provided by McCormick [5,6] with details on their size, geographic and chronological distribution, and their rural, urban, or military character. The author suggested that most of these features represent the victims of various epidemics such as Antonine, Justinian and/or Cyprian plagues that ravaged the Roman Empire during the Late Antiquity (ca 2^nd^-6^th^ centuries CE). Moreover, it was noted that about one third of the listed mass burials provide clear evidence of violent death [5], and only two sites characterized as violent death burials are located within the borders of the Roman Empire.

Mass burials and mass graves in general were not a customary way of interring the dead in the Roman Empire as these were mostly used during catastrophic events similar to the other periods [5,6]. During the Late Republic (200−27 BCE) and the Early Empire (27 BCE-180 CE), Roman burials mostly consisted of cremations, i.e., the cremated remains of the deceased were placed in various receptacles such as urns (ceramic, glass, metal) which were inserted in the grave. After the 2^nd^ c. CE, inhumation burials became increasingly common and completely predominant in the 3^rd^/4^th^ centuries CE. The deceased were usually laid in the plain ground or brick tombs, and only the wealthiest were placed in stone or lead sarcophagi [7].

In the Roman province of *Pannonia,* Osijek was one of the most important settlements located in the immediate vicinity of the Danube military border (*limes*). The Romans conquered the region during the 1^st^ c. BCE. After their victory in the Pannonian-Delmatian Uprising in 9 CE (the so-called *Bellum Batonianum*), they began implementing measures to consolidate their power by building military fortifications on the Danubian *limes*. An auxiliary camp was established in the immediate vicinity of the already existing Celtic-Pannonian settlement. Soon thereinafter, a Roman civilian settlement (*canabe*) developed near the camp. Due to its favorable location on the elevated southern bank of the Drava River, a bridge and a road intersection, *Mursa* became an important trade and craft center. The process of urbanization, which was initiated by Emperor Trajan, fully developed during the reign of Emperor Hadrian, who in 133 CE granted it the status of a colony (*Colonia Aelia Mursa*) [8]. As one of the largest settlements in the eastern part of the Roman province *Pannonia Inferior*, *Mursa* was the civilian and administrative center of this part of the military border zone. It continued to develop from the time of Hadrian until the Marcomannic Wars in the second half of the 2^nd^ century CE, when it was severely damaged. This was followed by a reconstruction phase under the Severan dynasty (193–235 CE), when the city again flourished and developed economically. The city played an important role in several historic events, especially in the 3^rd^/4^th^ centuries CE during the so-called ‘Crisis of the Third Century’ (235–284 CE) when various emperors and claimants fought for the throne resulting in numerous battles that significantly weakened the Empire. For example, in 260 CE emperor Gallienus fought against Ingenuus, and in 351 CE emperor Constantius II defeated Magnentius in battles that took place outside the walls of *Mursa* [9–11]. After the Gothic incursions in 378 CE, no significant reconstructions were undertaken, and with the Hunnic conquests in 441 CE, *Mursa* ceased to exist as an urban settlement [8,9].

**Fig 1:**
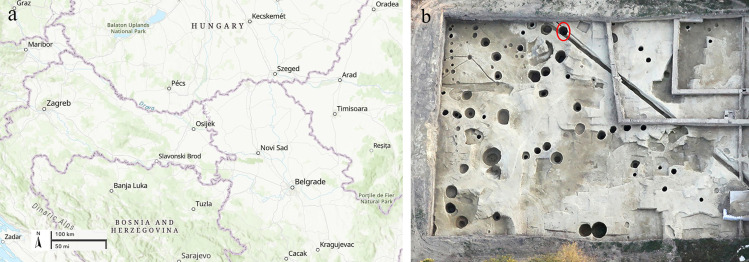
Map showing: (A) the geographic location of Osijek (base map credit: USGS National Map Viewer, https://apps.nationalmap.gov/viewer/); (B) the exact location of the well SU 233/234 during the excavation (in red circle).

Here we present new radiocarbon, bioarchaeological, isotopic and genomic data for an unusual burial feature (mass grave) recently excavated at the location of the University Library in Osijek, eastern Croatia (Roman period *Mursa*; Fig 1A). By describing this Roman period burial context and by presenting new cultural and biological information on the individuals interred in this mass grave we hypothesize that these are the victims associated with one of numerous catastrophic events occurring as a direct result of the ‘Crisis of the Third Century’, most probably the battle of *Mursa* from 260 CE.

## Materials and methods

### Radiocarbon dating

Direct radiocarbon dating of human bone/tooth samples was conducted at the Curt-Engelhom-Centre for Archaeometry, Mannheim, Germany (SK 2, SK 4, SK 5) and the HUN-REN Institute for Nuclear Research (HUN-REN ATOMKI), Debrecen, Hungary (SK 7).

### Bioarchaeological analysis

Conventional bioarchaeological analysis (sexing, ageing, metric and non-metric analysis, paleopathology) was conducted at the Laboratory for Evolutionary Anthropology and Bioarchaeology, Centre for Applied Bioanthropology, Institute for Anthropological Research, Zagreb, Croatia. The methods described by Buikstra and Ubelaker [12] and Klales [13] were used to estimate the sex and the age at death of the studied individuals. All individuals were thoroughly examined for the potential presence of different pathological conditions as described in Ortner’s [14], and Aufderheide and Rodriguez-Martin’s [15] identifications of pathologies. The stature was reconstructed based on the maximum humerus and femur length by using regression formulae proposed by Trotter [16].

In terms of injuries, all skeletons were examined macroscopically for the possible presence of trauma using methods proposed by Maples [17] and Lovell [18]. The location of the injury on the skeletal element was recorded, as well as the shape, dimensions, and any possible complications. A distinction was made between ante- and perimortem skeletal injuries. Antemortem injuries were identified by evidence of healing and bone remodeling [19], while perimortem injuries were identified with the following criteria: absence of healing and formation of new bone [19], fragments remaining attached to one another [19], internal beveling [20], defined or sharp edges [21], and flat or polished surfaces with macroscopically visible striations [22]. Considering that ground pressure and postmortem damage can mimic perimortem injuries [23], the differential texture and color of the lesions were used to differentiate between perimortem and postmortem trauma [19,20].

### C/N stable isotopes analysis

Collagen extraction for bulk *δ*^13^C and *δ*^15^N stable isotope analysis for the human samples considered in this study were conducted at the laboratories of the Centre for Applied Bioantropology, Institute for Anthropological Research, Zagreb, Croatia following the modified Longin protocol [24] with recommended modifications by Brown et al. [25], and Collins and Galley [26]. Between 355 and 555 mg of bone samples were first subjected to superficial mechanical abrasion, followed by treatment in 0.5M HCl until complete demineralization. Afterward, the samples were rinsed three times with purified water and gelatinized in a pH3 solution at 70ºC for 48 hours. The reflux-insoluble residues were removed using Ezee-Filter^TM^ separators. The supernatant was frozen for 24 hours before being freeze-dried in preparation for EA-IRMS analysis which was performed at Sercon Analytical Ltd, UK.

### Ancient DNA data

Sampling for ancient DNA analyses was performed inside the clean room facilities of the Archeo- and Paleogenetics group at University of Tübingen. Teeth were sampled by cutting at the enamel-dentin junction and drilling into the crown with a dentist drill. Petrous bones were sampled by drilling into the dense inner ear after removing a thin layer from the surface. DNA was extracted from bone/tooth powder and transformed into genetic libraries with the UDG-half protocol to reduce DNA damage [27,28]. The libraries were both shotgun sequenced and enriched for 1.24M SNPs across the human genome [29]. Data processing followed the EAGER pipeline (v1.92.55), which include adaptor trimming, sequence alignment to human reference genome, removal of duplicate sequences, and authentication control for post-mortem damage [30–33]. The data was trimmed for 2 bp on both ends to avoid DNA damage-related errors, and genetic sex was determined using X and Y chromosome coverage [34,35]. Contamination was assessed using ANGSD [36], schmutzi [37] and contamLD [38], while mitochondrial DNA and Y-chromosome haplogroups were determined using Haplogrep2 [39] and Yleaf [40], respectively. ANGSD-based estimates of X chromosome contamination in male individuals are considered reliable when more than 100 SNPs are available on the X chromosome. In the case of female individuals or when this threshold is not met, for petrous bones we relied on mtDNA contamination estimates generated by schmutzi, while for teeth contamLD was employed. We carried out the PCA as discussed in the main text using smartpca [41] (using “shrinkmode: YES”) from the EIGENSOFT package (v18140) and qpAdm/qpWave was conducted using the Admixtools2 software in R with allsnps option YES [42]. The following outgroup populations were used for qpAdm/qpWave: OldAfrica (KPL001, NYA002, NYA003, I0589.AG, I1048.AG, I10871.AG, I10872.AG, I10873.AG, I10874.AG), Russia_MA1_UP.SG, Iran_GanjDareh_N.AG, Israel_Natufian.AG, Turkey_Central_Pinarbasi_Epipaleolithic.AG, WHG (KO1_noUDG.SG, Loschbour.DG, LaBrana1_noUDG.SG), Mesopotamia_PPNA.AG, Morocco_Iberomaurusian.AG, Mongolia_EIA_SlabGrave_1.AG. Using these outgroups, qpWave analysis is conducted for every possible pair among Roman period individuals from *Mursa* as well as Early Iron Age individuals from Croatia separately (S1 File: qpWave). Using the same outgroups, we conducted distal qpAdm analysis using the five west Eurasian ancestral sources, mentioned in text. As for proximal models, we attempted one-way models with the grouped Croatia_EIA.AG to test for continuity. Next, we added a secondary source from a broad subset of ancient populations guided by the PCA position of each individual (two-way models). Finally, we also tested one-way models and report in Fig 7 those if they have a better statistical fit (p > 0.05) compared to the two-way models. All tested qpAdm models for each individual are reported in Supp Info 1. Genotype data for the source and outgroup populations are accessed from the Allen Ancient DNA Resource (AADR) v62 [43].

**Fig 2:**
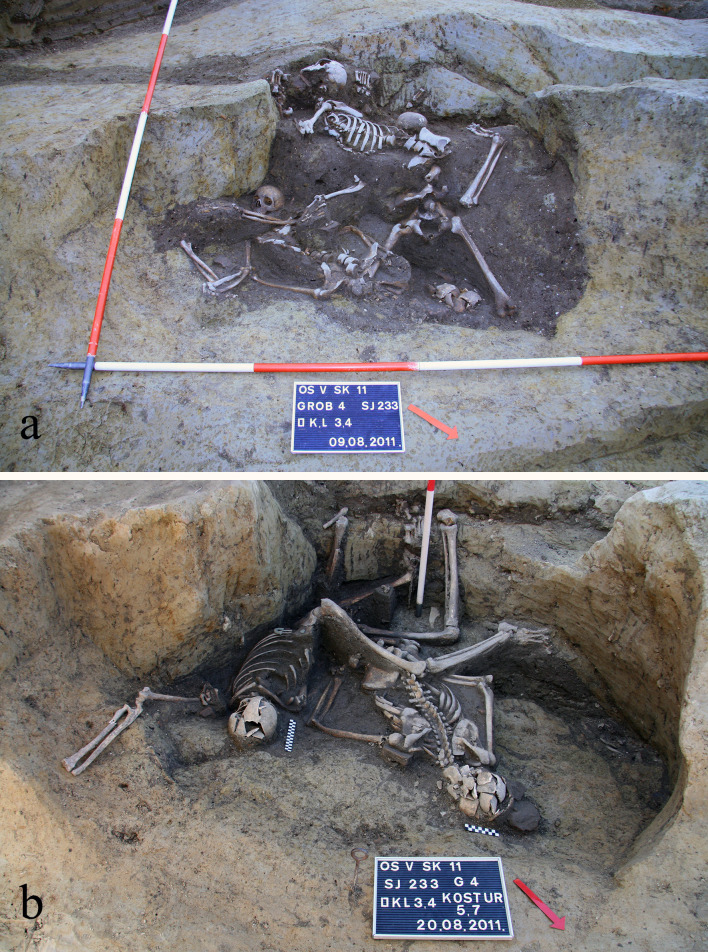
The well SU 233/234 during different excavation phases showing the position of the skeletons: (A) SK 2, SK 3 and SK 4; (B) SK 5 and SK 7.

**Fig 3:**
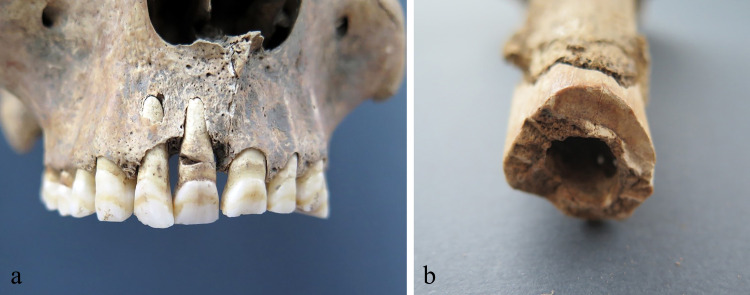
(A) Broken maxillary central right incisor; (B) cut to the diaphysis of the left humerus, detail, SK 4.

**Fig 4:**
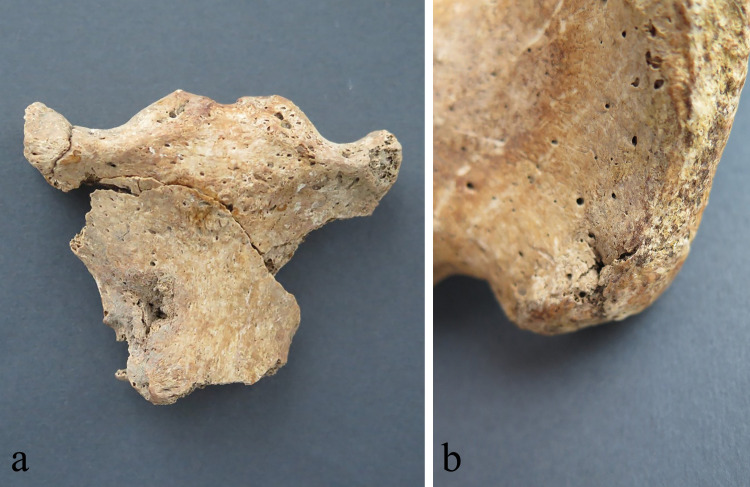
(A) Puncture wound on the anterior side of the manubrium, SK 4; (B) puncture wound on the posterior side of the right ilium, SK 5.

**Fig 5:**
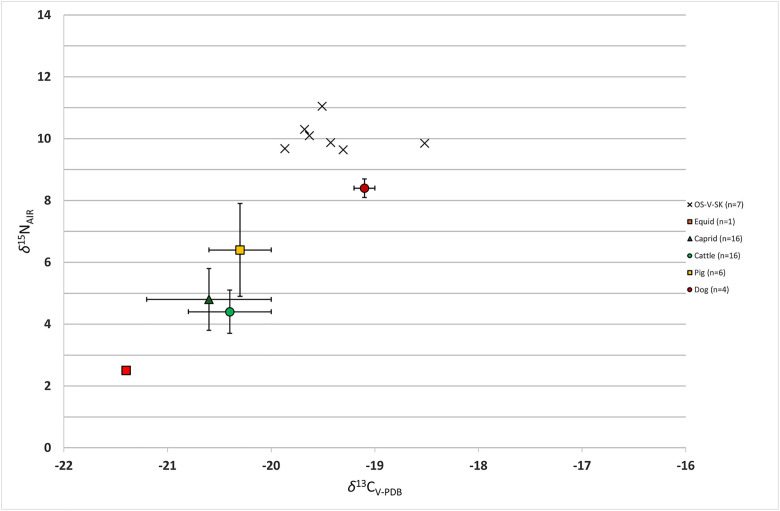
The chart showing C/N stable isotope values for the individuals from *Mursa* (the faunal baseline adapted from Lightfoot et al.).

**Fig 6:**
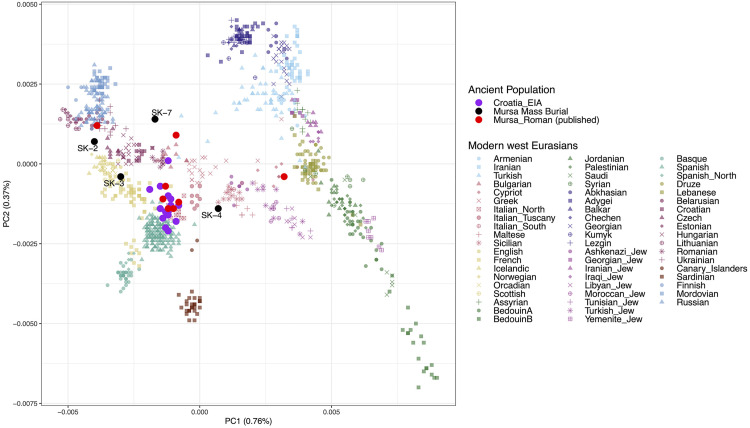
A West-Eurasian PCA projecting from newly generated and previously published ancient individuals from *Mursa* and Croatian Iron Age onto the modern-day genetic diversity.

**Fig 7:**
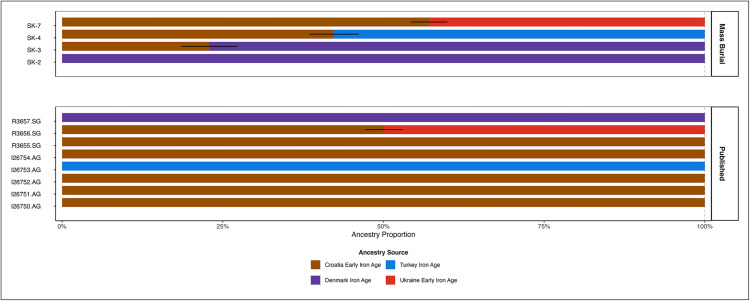
Modelling of ancient individuals from *Mursa* using proximal sources in qpAdm. The pre-Roman local ancestry is represented by Croatia Early Iron Age (Croatia_EIA.AG), the Eastern Mediterranean ancestry by Turkey Iron Age (Turkey_Aegean_Bodrum_Halikarnassos_Ancient1.AG), the Northern European ancestry by Denmark Iron Age (Denmark_IA.SG), and the Eastern European ancestry by Ukraine Early Iron Age (Ukraine_Dnieper_EIA.SG). These sources are chosen from a range of viable models and serve as representative population of the distinct ancestries found in *Mursa*.

### Ethics statement

No permits were required for the described study, which complied with all relevant regulations. The curator who excavated and is in charge of the remains is a co-author of the study and gave their permission to use the said remains for the analyses described in this section which complies with all relevant regulations. No ethical approval was required to study the human remains from *Mursa* (Osijek) as these are archaeological specimens 2,000 years old. The studied human remains are curated by the Archaeological Museum Osijek, Osijek, Croatia.

## Results

### Archaeology and absolute chronology

In 2011, protective archaeological excavations were carried out at the location of the future library of the Josip Juraj Strossmayer University of Osijek, located in the northern part of the university campus, i.e., the former ‘Drava’ barracks; coordinates 45°33′26″N 18°42′31″E (S1A–1B Figs). The largest number of recorded archaeological features is attributed to the water-wells of different depths with diameters ranging between 1.50 and 4.47 m, while the smaller number of features were pits of different shapes and depths (Fig 1B) [44]. The water-wells were filled with dirt after losing their primary function, and new ones were dug next to them. The number of wells found during the excavation indicates their importance in supplying the inhabitants of *Mursa* with water. Small archaeological finds preliminarily date the site to the 2^nd^/3^rd^ century CE [44]. The well SU 233/234 (G 4) was a well dug in the plain ground with a diameter of 2 m and depth of about 3 m. The complete remains of seven adult individuals were found in this used water-well that was repurposed for the grave (Figs 2A–2B; S2A–2B Figs); the skeletons were found articulated, in different positions and in different depths (SK 1 at the top, and SK 7 at the bottom of the feature). Four direct radiocarbon dates–one from the top (SK 2) and one from the bottom (SK 7) and two in between (SK 4 and SK 5)–place the burial to the second half of the 3^rd^ and the beginning of the 4^th^ century CE (SK 2: MAMS-68836, 1783 ± 18 BP, 232–335 cal CE; SK 4: MAMS-73951, 1748 ± 19 BP, 244–375 cal CE; SK 5: MAMS-73944, 1765 ± 19 BP, 239–346 cal CE; SK 7: DeA-43762, 1770 ± 15 BP, 240–340 cal CE) (S1 File: Radiocarbon dates). Radiocarbon dates agree with a coin found in the fill of SU 233/234 approximately at a depth of 1.5 m – a Roman *sestertius* minted in *Viminacium* during the rule of emperor Hostilian in 251 CE (S3A–3B Figs). There is no evidence to indicate that the burial was disturbed by subsequent activities, either human or animal.

### Demography and general health

All seven skeletons (SK 1-SK 7) from the well SU 233/234 belong to adult males: four are younger adults (between 18 and 35 years), and three are middle-aged adults (between 36 and 50 years). All individuals were robust, and their average stature was around 172.5 cm ranging between 167 and 176 cm. The skeletons exhibit a certain number of pathological changes such as vertebral osteophytosis, Schmorl’s nodes, dentoalveolar disease (antemortem tooth loss, caries and alveolar abscess), pronounced entheseal changes on the upper and lower limbs, and indicators of early-life stress (enamel hypoplasia, *cribra orbitalia*, porotic hyperostosis) (S1 File: Demography, pathology, stature). All individuals show evidence of active new periosteal bone formation on the visceral side of the ribs (S4A–4B Figs). Beside these changes, there are several examples of healed and unhealed antemortem injuries: SK 1 (20-25y) exhibits rounded (7 mm in diameter), very shallow, antemortem healed blunt force trauma with remodeled edges on the right side of the frontal bone (located approximately 44 mm superior of the right orbit); SK 4 (32-42y) exhibits antemortem unhealed fracture of the 6^th^ left and the 8^th^ right rib (S5 Fig); SK 5 (35-45y) shows oval-shaped, very shallow, antemortem healed blunt force trauma on the left side of the frontal bone (located approximately 34 mm superior of the left orbit) with the dimensions of 5 x 3 mm. And finally, two skeletons show perimortem injuries sustained by different types of a weapon/tool. SK 4 (32-42y) exhibits: (i) a puncture wound on the anterior side of the manubrium that completely pierced the bone (on the anterior side the dimensions of the injury are 8 x 5 mm with indented edges while on the posterior side the dimensions are 3 x 3 mm with a bone fragment projecting toward the lungs); (ii) broken maxillary central right incisor with the main impact on the anterolateral side of the root; (iii) a cut on the medial side of the diaphysis of the left humerus that penetrated into the bone approximately 7 mm almost reaching the medullary cavity (the cut is polished with the dimensions of 19 x 3 mm followed by active inflammation superior and inferior of the cut); (iv) a cut on the tubercle of the 9^th^ right rib that split the tubercle in half (Figs 3A–3B, 4A; S6A–6B Figs). SK 5 (35-45y) reveals a puncture wound on the posterior side of the superior iliac spine of the right ilium that did not pierce the bone completely – the dimensions of the injury are 2 x 2 m with the indented edges and without any signs of healing and/or inflammation (Fig 4B).

### Carbon/nitrogen stable isotopes

The samples for C/N stable isotopes analysis were taken from the 7^th^ left rib of each individual to avoid the possibility of using two samples from the same skeleton and to obtain a comparable measurement. All seven samples provided collagen of good quality with C:N ratios falling between 3.1 and 3.2 [45,46]. The values of both carbon and nitrogen for all individuals are broadly uniform and fall in the range between −19.9 and −18.5‰ for *δ*^13^C with a mean value of −19.4‰ (± 0.6), and between 9.6 and 11.0‰ for *δ*^15^N with a mean value of 10.1‰ (± 0.5) (S1 File: C/N stable isotopes). Fig 5 shows that the isotopic values are indeed uniform for all individuals (the faunal baseline is adapted from Lightfoot et al. [47]). One individual (SK 7) exhibits a more positive shift in ^13^C values, which can imply an additional intake of C_4_/marine component in their diet while the individual SK 2 shows increased ^15^N values, yet with the lesser amount of C_4_ plant component.

### Genomic data

We screened either a tooth or a petrous bone from each of the seven individuals for ancient DNA (aDNA) analysis using shallow shotgun sequencing. All except SK 1 yielded sufficient endogenous DNA (> 0.1%) paired up with the typical aDNA damage to undergo a targeted enrichment approach known as “1240K Capture” [29]. As a result, we generated genome-wide data for six individuals (SK 2–7). After the estimation of DNA contamination in the newly generated data, SK 5 and SK 6 did not pass our quality filters (see Materials and methods). Consequently, we proceeded with downstream analyses using the four remaining individuals (SK 2, SK 3, SK 4, and SK 7). We genetically sexed all the individuals as male, corroborating the anthropological findings (S1 File: aDNA Data Review).

We carried out a Principal Component Analysis (PCA) by projecting the genomic data of the ancient *Mursa* individuals onto the genetic diversity of 60 modern west Eurasian populations [48]. As reference, we also projected the published Iron Age individuals from present-day Croatia [49,50], along with eight other Roman individuals from a different archeological context in *Mursa*, whose genome-wide data have been previously reported [49–51] (Fig 6).

In the PCA, all newly sequenced individuals from the *Mursa* mass burial display a high genetic diversity. On the other hand, the Croatian Iron Age genetic background shows a largely homogenous cluster. We corroborate this genetic heterogeneity using qpWave [52] by showing that none of the individuals in this archaeological context can be modeled in genetic continuity with the local Early Iron Age population (S1 File: qpWave). This contrasts with eight published Roman individuals from the same city, the majority of whom show PCA placements and qpWave results consistent with the local Iron Age variation (though with three outliers). The high diversity among the tested mass grave individuals is further reflected by the fact that SK 2, SK 3, SK 4, and SK 7 belong to the distinct paternal haplogroups N1a1, R1b1, I2a1b, and I1a3a1, respectively (S1 File: aDNA Data Review). Despite this large genetic heterogeneity, we can observe a general trend with SK 2, SK 3, SK 7 and two published outliers (R3656.SG and R3657.SG) plotting higher along PC2 than the proceeding Iron Age group, in proximity of present-day Northern and Eastern European populations. In contrast, SK 4 is shifted to the right along PC1 and projects on-top of modern-day Sicilians. This shift towards eastern Mediterranean populations has been previously described for Roman-associated individuals from the Italian, Iberian and the Balkan peninsula [53–57]. Notably, one of three previously published genetic outliers from *Mursa* (R3653.SG) exhibits a PCA placement in close proximity to present-day Near Eastern populations.

Finally, we modeled the four mass burial individuals, along with the other published Roman genomes from *Mursa* using qpAdm [58] (Fig 7). Following [55], we first attempted to quantify the five distal genetic components for the ancient individuals using Caucasian Hunter Gatherers (CHG), Eastern Hunter Gatherers (EHG), Iron Gates Mesolithic, Levant Neolithic, and Turkey Neolithic individuals. Individual components of these distal ancestries varied greatly for each individual, confirming the large genetic diversity found in the mass grave and more generally in the Roman population of *Mursa* (S1 File: Distal qpAdm; S7 Fig). In addition, and in line with the PCA and qpWave findings, none of the newly sequenced individuals could be modelled in qpAdm using the Croatian Iron Age population as the only source, while five of the published individuals fit this model successfully (S1 File: 1-way Proximal qpAdm). We then attempted to model the mass burial individuals and the previously published genetic outliers as a mix between the local gene-pool and other ancient ancestries of related periods, guided from the direction of their shift in PCA space. SK 3 required a contribution from a Northern or Central European source, modelled as Iron Age populations from Denmark [56], Sweden [56], Norway [56], Northern Germany [59,60] and Poland [51], and ranging between 50 and 78% (S1 File: qpAdm SK 3). Instead, SK 2 and R3657.SG can be better modelled as fully deriving from similar Northern/Central European sources (S1 File: qpAdm SK 2, qpAdm R3657.SG). For SK 7 and R3656.SG, a two-way admixture model between the local Croatian Iron Age ancestry and Eastern European sources was required (42–66%), with multiple statistical fits for Iron Age populations from the Pontic Caspian region such as modern-day Ukraine [61], western Russia [62] or Kazakhstan [61–63] (S1 File: qpAdm SK 7, R3656.SG). Finally, SK 4 is best modeled using a two-way approach, with one ancestry deriving from the local Croatian Iron Age and a secondary contribution from a Eastern Mediterranean source, either from Western Anatolia [50] or mainland Greece [50] (48–60%) (S1 File: qpAdm SK 4). Interestingly, the published I26753.AG individual can be better modelled as fully deriving from a Near Eastern source, suggesting the presence of putatively first-generation migrants from the Eastern Mediterranean in *Mursa* (S1 File: qpAdm I26753.AG).

## Discussion

In this study we provide new insights about the victims of a catastrophic event occurring in the second half of the 3^rd^ century CE near Roman period *Mursa* by presenting new radiocarbon, bioarchaeological, isotopic and genomic data on the individuals recovered from a mass grave inside a water-well located just outside of the walls of *Mursa*. To better understand this rare find we contextualized the presented information with the available archaeological data as well as known historic sources.

The available archaeological data suggest that the site of the University Library in Osijek was in use for various purposes during the 2^nd^/3^rd^ century CE. The archaeological object in question (SU 233/234) represents a used water-well where the remains of seven individuals were interred (most probably thrown in) before the well was filled with soil. The vertical stratigraphy of the whole feature as well as the position of the skeletons strongly indicate that this was a single episode rather than a recurring deposition over a longer period. This hypothesis is also supported by the fact that four direct radiocarbon dates (from the top, middle and bottom of the well; SK 2, SK 4, SK 5 and SK 7) are almost identical pointing to the second half of the 3^rd^ century CE. Furthermore, all skeletons were found completely articulated indicating that the individuals were thrown into the well while their bodies were still fully fleshed, and not partially or completely skeletonized, suggesting a very short time between their death and the moment of interment.

Most of the 3^rd^ century CE in the Roman Empire was characterized by the so-called ‘Crisis of the Third Century’ that ended with Diocletian’s reorganization of the Empire [64]. During this crisis, numerous battles fought between various claimants to the throne, significantly weakening the Empire. One such battle took place in the immediate vicinity of *Mursa* in 260 CE when the emperor Gallienus defeated the claimant to the imperial throne Ingenuus. Although very few details about this battle are known and the total number of casualties cannot be reconstructed with certainty, available contemporary sources state that during the battle many Roman soldiers have perished [9,65] with Ingenuus either being killed by his own soldiers or drowning himself in the river after the battle [65]. Slightly more information is available for the second battle of *Mursa* that took place in 351 CE between the emperor Constantius and the usurper Magnentius. According to the contemporary sources it was one of the bloodiest battles in the history of the Roman Empire with supposedly 30,000 killed on Constantius’ side that was victorious and 24,000 killed on Magnentius’ side [9,66]. However, it is not known what happened to the victims of both battles of *Mursa* as written sources did not provide any information on this topic and archaeological features that could be associated with these battles have been nonexistent so far. The deceased from both sides that probably numbered in hundreds and even thousands could have been buried in mass graves or burnt on huge pyres to prevent the spread of diseases but could have also been dumped in the nearby Drava River. All four direct radiocarbon dates from the mass grave are consistent with the battle of *Mursa* in 260 CE rather than the one taking place in 351 CE. Historic sources mention that after the battle of *Mursa* in 260 CE, Gallienus showed no clemency towards Ingenuus’ supporters and it would appear that mass executions were not only limited to prisoners of war [67]. In this context, the presented data open the possibility that the individuals from the mass grave in Osijek were soldiers, possibly representing the victims of the battle of *Mursa* in 260 CE whose remains were interred in a well outside of the city walls after the battle. There is only one known case that slightly resembles the example from Osijek and that is the find of the complete skeleton of a Roman soldier in a well from Velsen I in Northern Netherlands – the individual was presumably a victim of a violent act, and his body was disposed of in a water-well and was found below the deposition of stones, pottery and domestic refuse [68,69].

Another possibility should be accounted for. Namely, the losses incurred by both loyalists and rebels at the battle of *Mursa*, as well as the ensuing large-scale retaliation, must have critically weakened the frontier garrisons, a detail which could not have escaped the attention of Rome’s Danubian neighbors [67]. It seems that during the same year (260 CE), the invasion led by the Sarmatians and Quadi targeting *Pannonia* inflicted grievous losses and was a major disaster for the whole province [70]. Although unambiguous archaeological data about destruction layers dated to that period in the southern part of *Pannonia Inferior* (the region around *Mursa*) is absent [67], the possibility that the victims of these incursions have been interred in the water-well from Osijek must be taken into consideration.

Demographic characteristics of the skeletal assemblage from Osijek are rather unusual as these remains represent seven younger/ middle-aged males. This is significantly different from demographic characteristics of attritional (normal) mortality assemblages as well as those catastrophic assemblages associated with epidemics, indiscriminate massacres and/or famine [71–77]. However, the demographic composition of the Osijek sample is almost identical to those seen in battle-related assemblages such as Tollense Valley [78], Himera [79] and Aljubarrota [80], a mass grave from Vilnius containing the remains of soldier’s from Napoleon’s Grand Army [81] or the one from Skopje (*Scupi*) probably representing a mass execution of soldiers [2,5]. Considering that in all these sites (as in Osijek) younger males are predominant with a complete absence or a very small percentage of women and children being present, the main hypothesis is that the individuals from *Mursa* were active soldiers. During the Roman period, approximately two-thirds of all soldiers (legionaries) enlisted between ages 17 and 20, while most of the others signed on between ages 21 and 25 [82]. Starting from the late 1^st^ c. CE, legionaries were generally expected to serve for 25 years [82]. Therefore, according to these criteria all individuals from Osijek fall into the category of ‘soldiers’. Moreover, the 4^th^/5^th^ century CE historian Vegetius stated that the recruits for the Roman army had to be between 1.70 and 1.77 m tall, but shorter men of good build could also be accepted since a strength was more important to a soldier than just a height [83]. In Osijek, all individuals (apart from SK 3) were over 1.70 m (average 172.5 m) which is much higher than the average height of males (168.6 m) recorded in the province of *Pannonia* during this period [84]. Besides, all individuals but one (SK 6) have pronounced entheseal changes on the upper and lower limbs as well as numerous Schmorl’s nodes on the vertebrae (apart from SK 4) indicating intense use of certain muscles, long-term physical activity, and mechanical load of the spine [85,86]. All these features suggest that the individuals from the Osijek mass burial were indeed soldiers.

The presence of new periosteal bone formation on the visceral side of the ribs of all seven individuals suggests a widespread infection while the unhealed state of these changes in all cases indicate that the *Mursa* individuals suffered from some kind of pulmonary disease during the final days of their lives. The presence of new periosteal bone formation in that skeletal location has been used as evidence for lower respiratory tract disease in the archeological record [87]. Numerous authors propose that fluid/pus accumulation and subsequent inflammation within the pleural cavity, usually resulting from lower respiratory tract diseases, can stimulate inflammatory periosteal reaction on the visceral side of ribs [88,89]. The most common respiratory infections to cause pleural inflammation of the ribs in modern populations include tuberculosis, pneumonia, and actinomycosis [90], thus some of these infectious diseases have to be considered also in this archeological context.

The most obvious paleopathological feature of the *Mursa* skeletal assemblage is the presence of ante- and perimortem injuries in three skeletons. Two antemortem cranial blunt force injuries on the frontal bones of SK 1 and SK 5 strongly indicate intentional violence as the main cause since this type of injury is usually associated with a face-to-face combat [91–93]. Additionally, the location of both of these injuries well above the so-called hat brim line suggests their violent nature rather than accident as the probable cause [94]. The presence of perimortem sharp-force trauma and puncture wounds in SK 4 and SK 5 are definite proofs of intentional violence. The morphology of these injuries strongly suggests that puncture wounds were probably caused by an arrow or a spear tip: the appearance of the entrance/exit wound on the manubrium of SK 4 is typical of high-speed projectile weapons such as arrows [95,96] while the dimensions and the appearance of the hip injury of the individual SK 5 suggests a weapon with rounded or square cross-section rather than the weapon with a flat blade such as a sword. However, due to the obvious challenges when differentiating between these classes of weapons in archaeological contexts it is impossible to determine with certainty the exact weapon in each case. On the other hand, the cuts on the humerus and the rib of SK 4 were caused by a long-bladed weapon (probably *spatha*) [95]. In the case of SK 4 everything points to a face-to-face combat as all perimortem injuries are located on the anterior side of the skeleton, while in the case of SK 5 the position of the injury suggests that the attacker was located behind the victim. Finally, the presence of multiple injuries in SK 4 and SK 5 provides further confirmation of intentional violence [97], probably sustained through multiple episodes, additionally confirms the hypothesis that during their lifetime these individuals served as soldiers.

Carbon and nitrogen stable isotope analyses provide a direct insight into dietary patterns and subsistence strategies of past populations. For example, carbon isotope values (*δ*^13^C) primarily reflect the types of plants consumed, distinguishing between C_3_ plants (e.g., wheat, barley) and C₄ plants (e.g., millet) or can differentiate marine from terrestrial resources [98,99]. Nitrogen isotope values (*δ*^15^N) reveal trophic levels and it is used to identify the relative contributions of protein sources in the diet [98,99]. All seven individuals from Osijek are tightly clustered and show almost uniform isotopic values of both carbon and nitrogen suggesting a very similar diet during the last few years of their lives. Combined C/N isotope values in these individuals indicate that they likely consumed a mixed C_3_/C_4_-based vegetal diet (e.g., wheat, vegetables, millet, etc.) with limited amounts of terrestrial animal protein and an even lower consumption of marine protein. Both *δ*^13^C and *δ*^15^N values are within the range of values presented in the study by Lightfoot et al. [47] showing that the Roman period on the territory of modern-day Croatia is associated with a limited, but isotopically detectable amount of marine consumption. While the samples from that study comprised individuals from coastal sites, a similarity in values can be seen for the skeletons presented here.

To date, no attempt has been made to reconstruct the diet of Roman soldiers by the means of C/N stable isotopes analysis, thus a direct comparison of our results with other similar studies cannot be performed. However, written sources state that the staple food of Roman soldiers was wheat. The rations of wheat were usually supplemented with meat (mostly salted pork), olive oil, wine, salt, lentils, and vinegar [82]. As the army was in the field for a longer period, this diet was usually supplemented and diversified with more protein-rich vegetables like beans [100]. The isotopic values from the Osijek individuals conform well to the available historic information on the everyday diet of Roman soldiers and furthermore suggest a military character of this assemblage.

Based on the aDNA analyses conducted on four individuals from the burial site, none were found to carry the local Iron Age genetic profile even though a group of Roman individuals from *Mursa* excavated in other contexts showed complete continuity with it. Two non-local ancestries found in the mass grave, which we model using various proxy sources from Iron Age populations located either in Northern/Central or Eastern Europe, are not uncommon in Late Roman contexts (ca 293–476 CE) from the Balkans [55]. Its presence in a military setting aligns with historical accounts of Late Roman armies, which frequently incorporated ethnically diverse groups such as Sarmatians, Saxons, and Gauls [101]. One among many possibilities is that the individual with Eastern European ancestry from Osijek could be descendant of the original Sarmatian soldiers defeated by the Roman Empire as witnessed by the Roman historian Cassius Dio [102]. Defeated Sarmatians were then drafted into the Roman army and posted as far as Britain, an event also supported by recent paleogenomic research [103]. In contrast, SK 4 exhibits a genetic profile carrying a Near Eastern influence as previously observed for several populations across the Roman Empire.

There is another mass burial from Osijek, located quite close to the one presented here and dated to the mid-3rd century CE, containing multiple commingled adult male skeletons with perimortem injuries that can also be associated with the battle of *Mursa* in 260 CE. However, ancient DNA analysis for this context is still underway so we cannot speculate about the genetic profile of these individuals and determine whether they show a more local genetic profile than the individuals from the well SU 233/234. At the moment, we still cannot say with certainty what does the ‘local’ genetic profile of *Mursa* in the 3^rd^ century CE looked like, but the existing genomic information indicate that most of the Roman period inhabitants of the city were consistent with the preceding local Iron Age variation with some outliers.

The coexistence of highly diverse genetic backgrounds among the genetically analyzed individuals and with the preceding population, strengthens the interpretation of these burials having militaristic origins. In fact, the observed genetic diversity might reflect the reliance of the Roman Empire on heterogeneous military recruitments, corroborating historical evidence for the integration of ‘foreign’ groups into imperial forces.

In conclusion, multidisciplinary work and close collaboration between researchers from various fields can help to gain a more comprehensive view of historically relevant events. The study of a mass grave from *Mursa* is an excellent example of such an approach as it revealed previously unknown information of the catastrophic consequences of the ‘Crisis of the Third Century’ in this part of the Roman Empire.

## Supporting information

S1 Fig The location of: (A) the site of the University Library in Osijek today (in red circle) (base map credit: USGS National Map Viewer, https://apps.nationalmap.gov/viewer/); (B) the site during the excavation (marked in number 5). (TIF)

S2 Fig The well SU 233/234: (A) at the beginning of the excavation showing the position of SK 2 and SK 3; (B) after the excavation. (TIF)

S3 Fig Roman *sestertius* minted in 251 CE in *Viminacium* during the reign of emperor Hostilian: (A) obverse: bust of Hostilian viewed from the right surrounded by the inscription C VAI HOST M QVINTVS CAE; (B) reverse: a woman standing, holding a branch in the right hand, and a globe in the left with a bull and a lion standing at her feet; the inscription PMSC – OLVIM at the top and AN XII on the bottom. (TIF)

S4 Fig Active new periosteal bone formation on the on the visceral side of the ribs: (A) SK 2; (B) SK 7. (TIF)

S5 Fig Antemortem unhealed fracture of the 6^th^ left rib, SK 4. (TIF)

S6 Fig (A) Puncture wound on the manubrium, posterior view, SK 4, (B) cut to the diaphysis of the left humerus, SK 4. (TIF)

S7 Fig qpAdm modelling of Roman individuals from *Mursa* using distal ancestral sources. Sample SK 7 could only be modeled with a negative value for one component and is therefore not depicted in this figure (addition information in S1 File: Distal qpAdm).
(TIF)

S1 File The spreadsheet containing additional demographic/ pathological, radiocarbon, isotopic and genomic information about the newly analyzed individuals from *Mursa.* (XLSX)
