# Supplementary figures and images for: Multidisciplinary study of human remains from the 3rd century mass grave in the Roman city of Mursa, Croatia
Source: PLoS One. 2025 Oct 15;20(10):e0333440. doi: 10.1371/journal.pone.0333440 (PMC12527192; doi:10.1371/journal.pone.0333440)

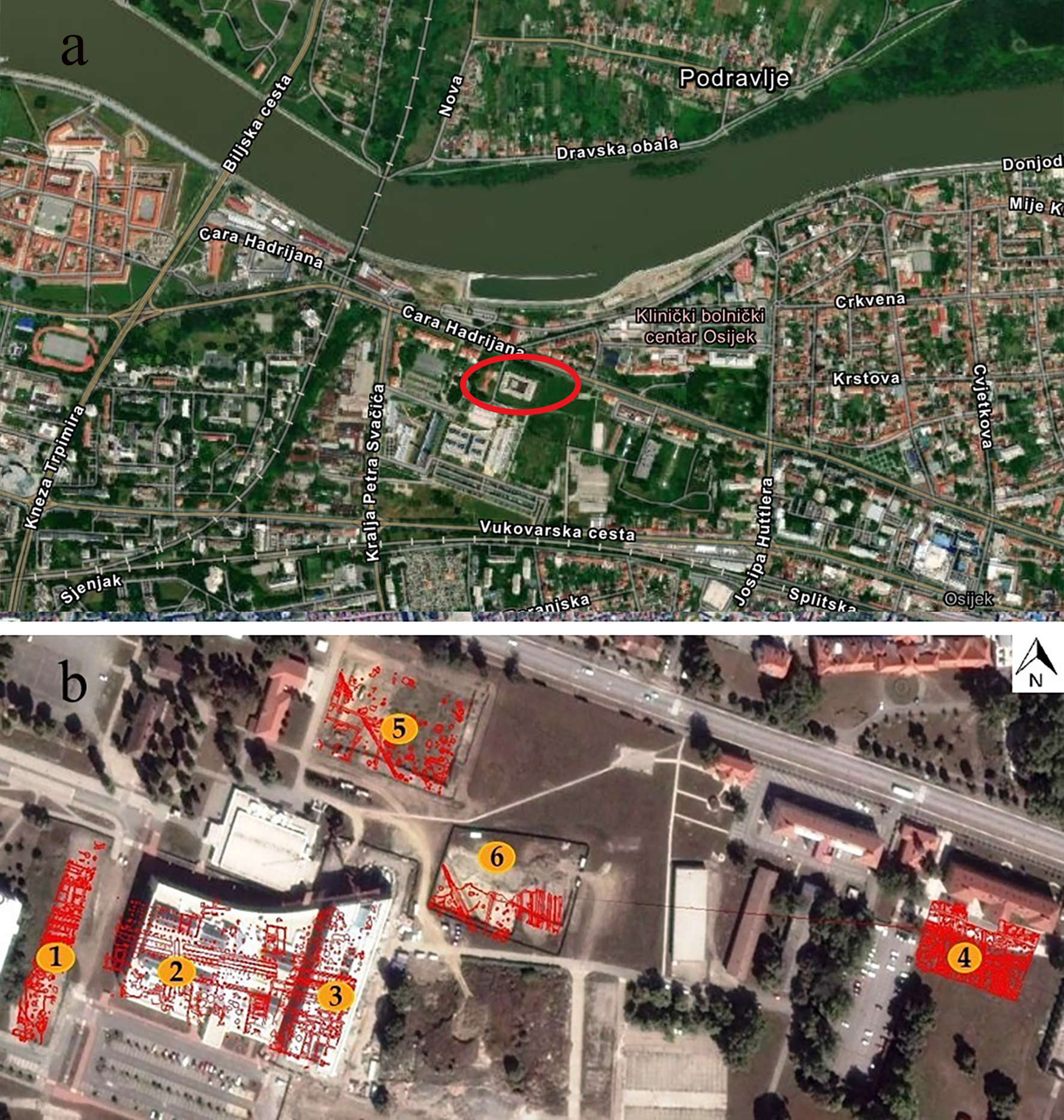

Supplement: S1 Fig — (TIF) [file pone.0333440.s001.tif]

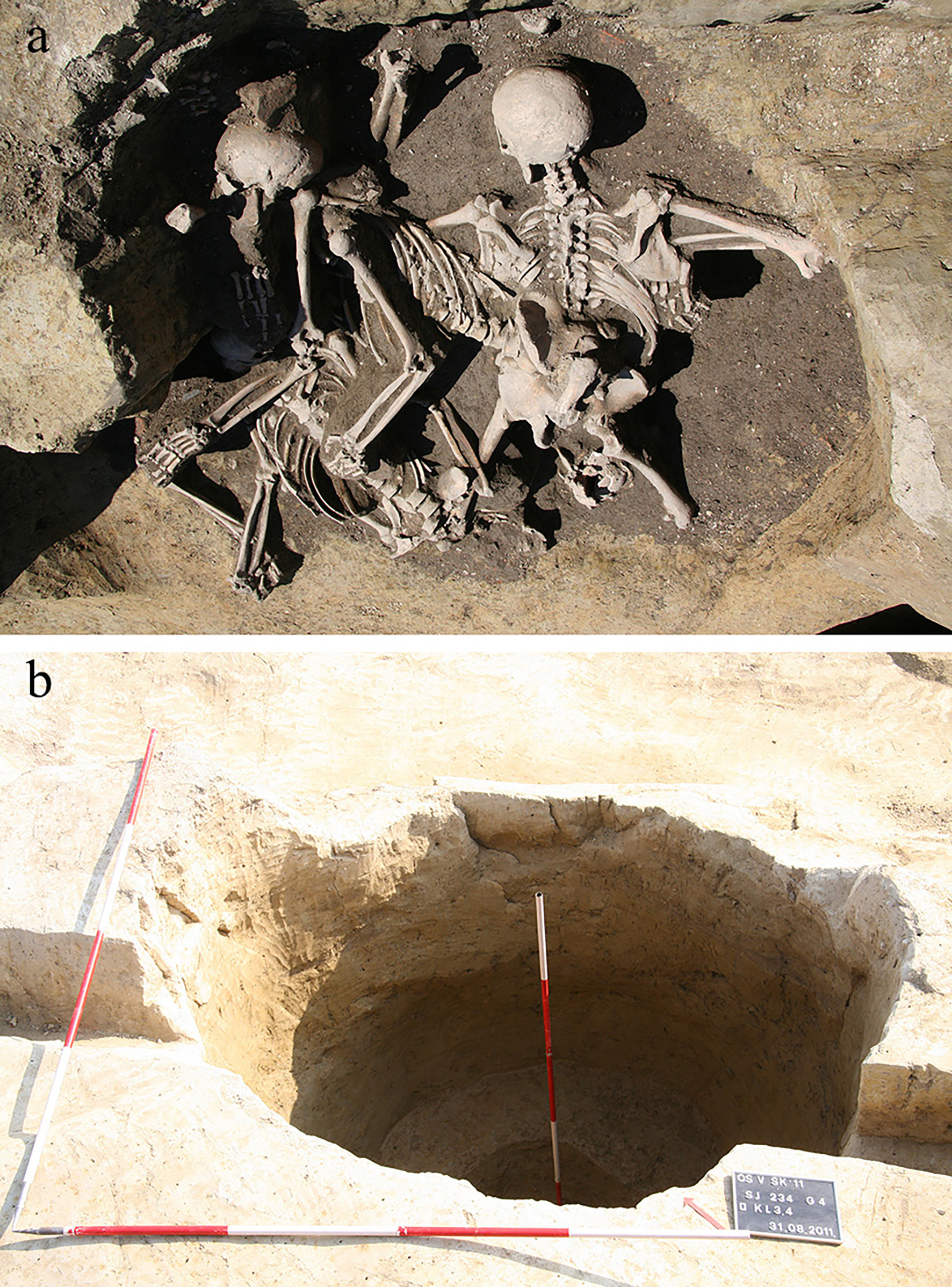

Supplement: S2 Fig — (TIF) [file pone.0333440.s002.tif]

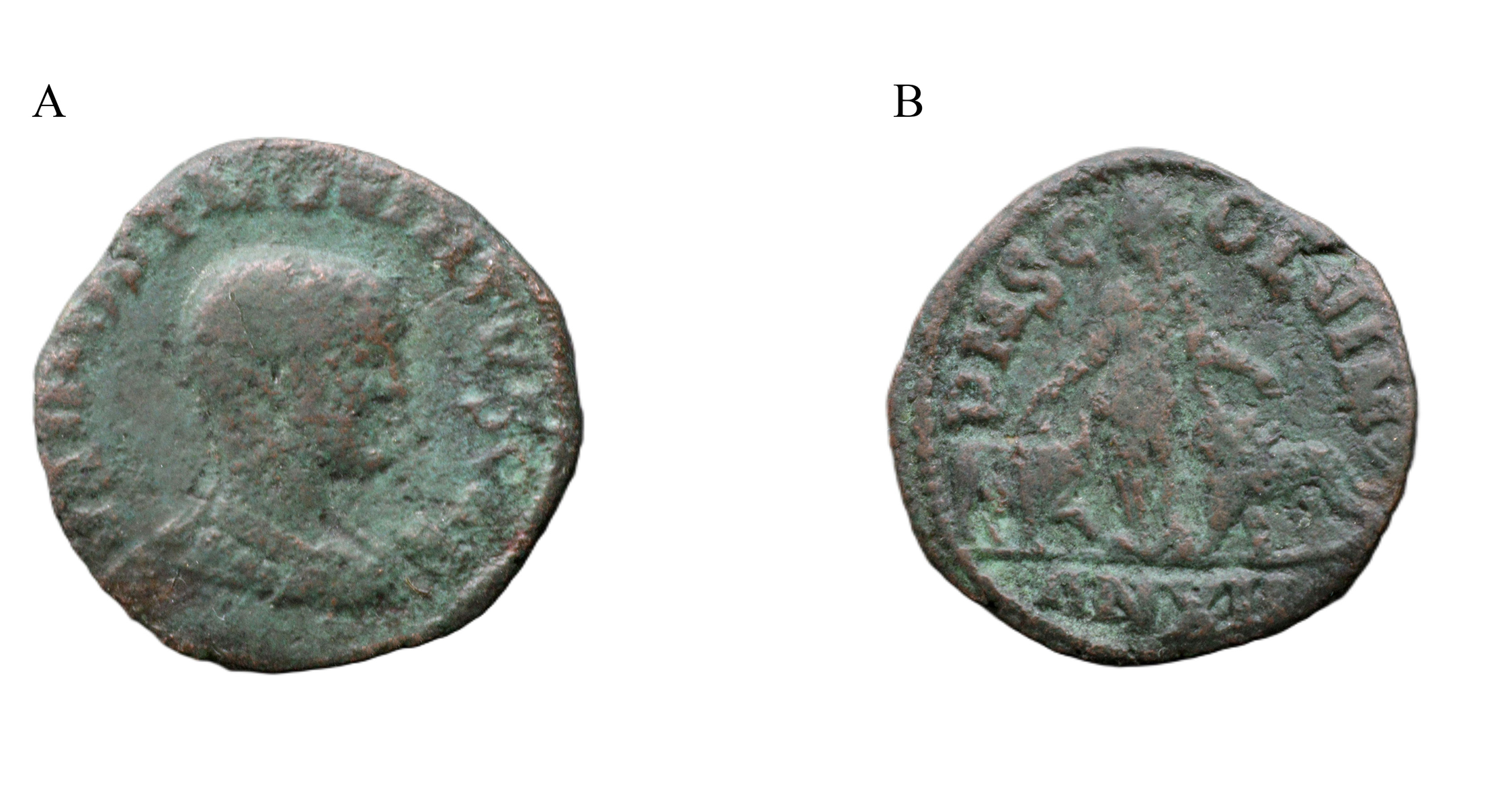

Supplement: S3 Fig — (TIF) [file pone.0333440.s003.tif]

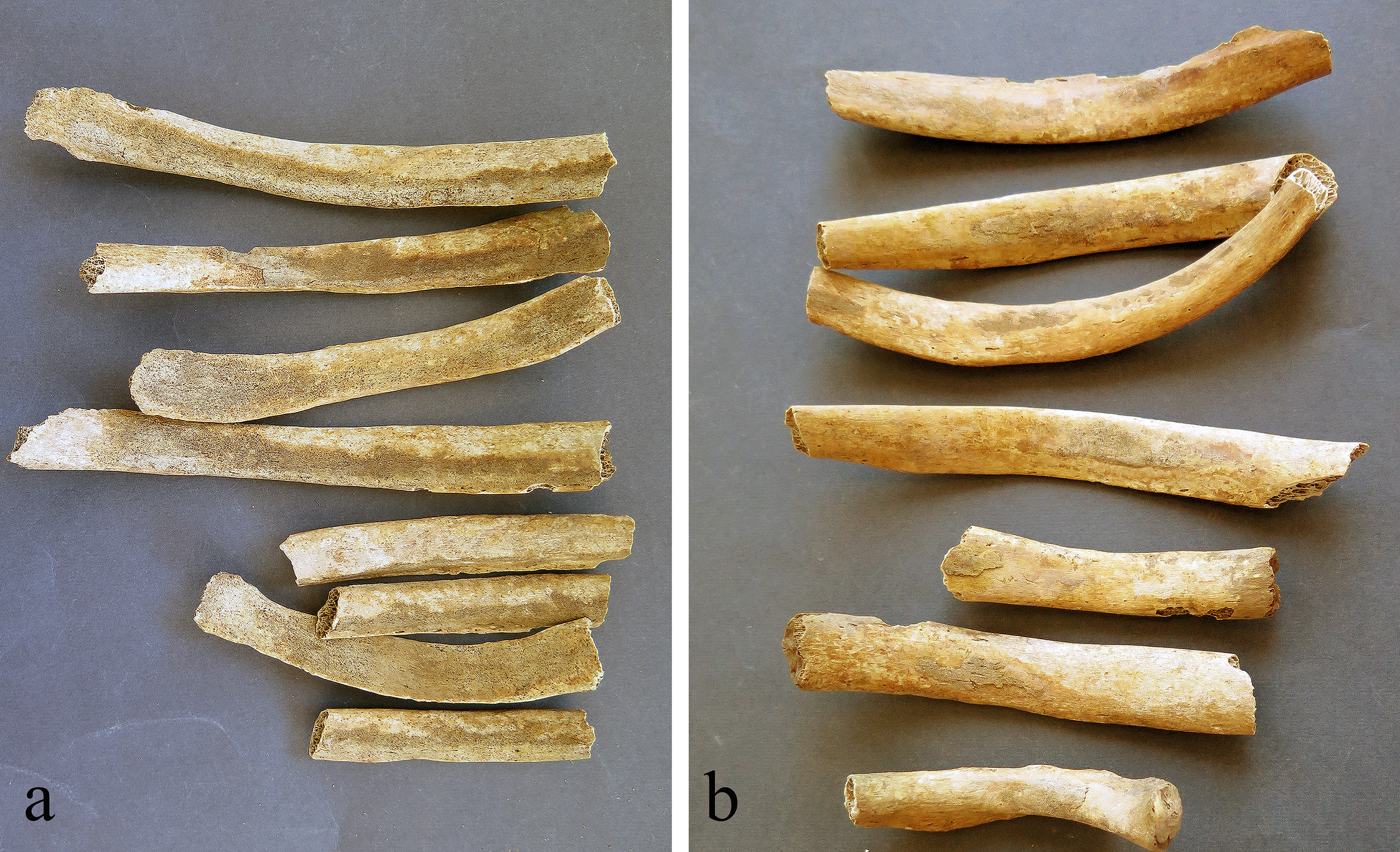

Supplement: S4 Fig — (TIF) [file pone.0333440.s004.tif]

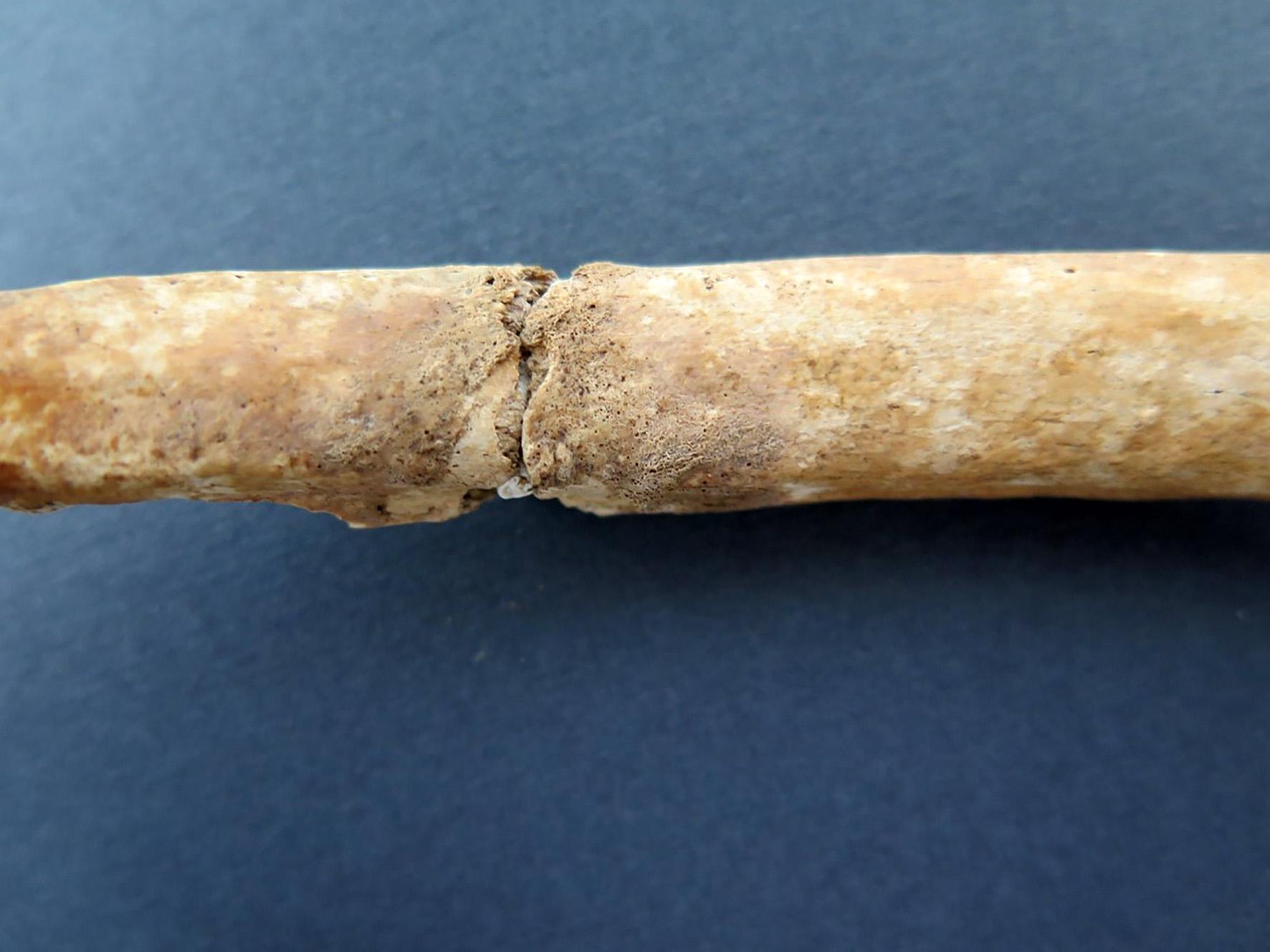

Supplement: S5 Fig — (TIF) [file pone.0333440.s005.tif]

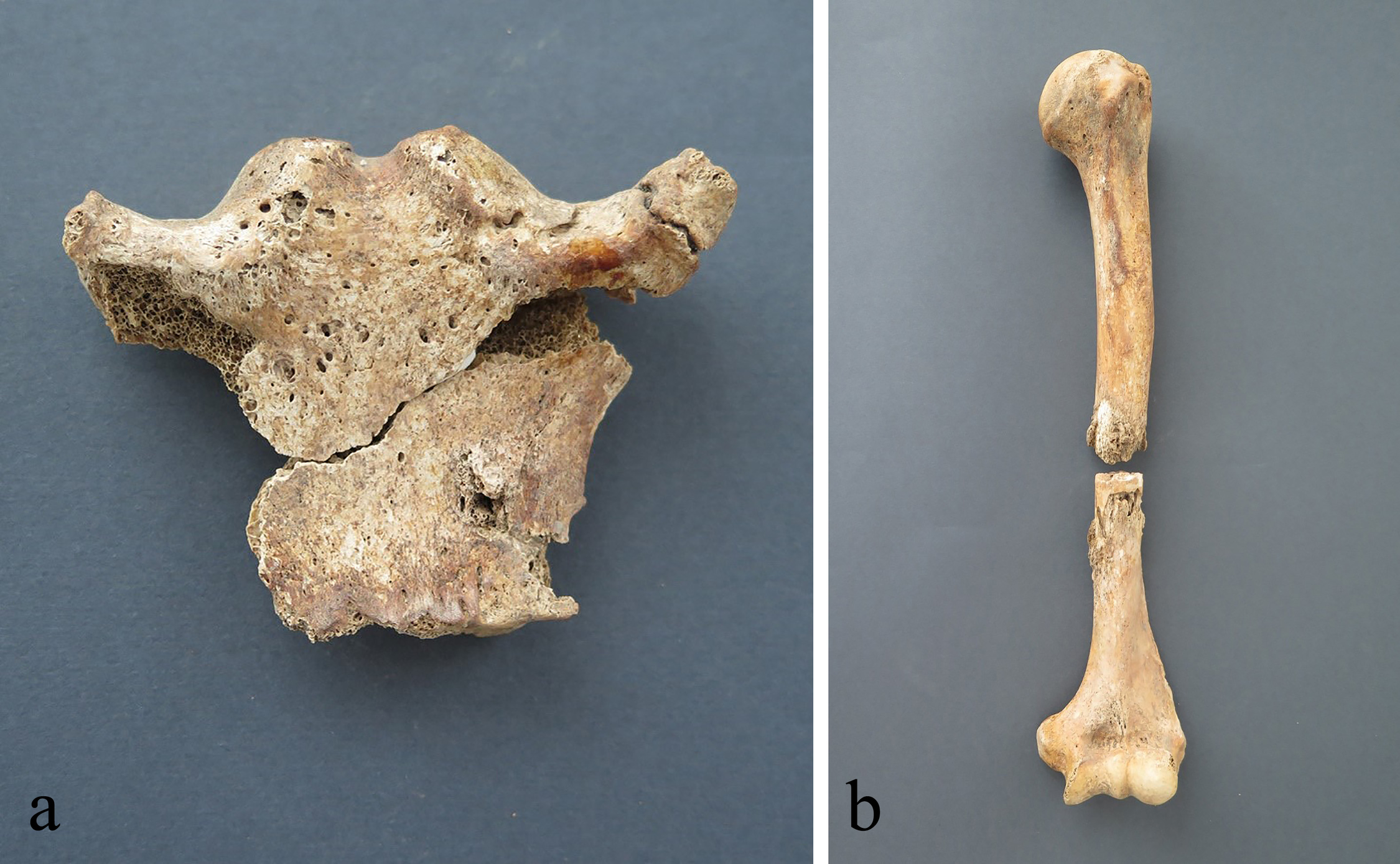

Supplement: S6 Fig — (TIF) [file pone.0333440.s006.tif]

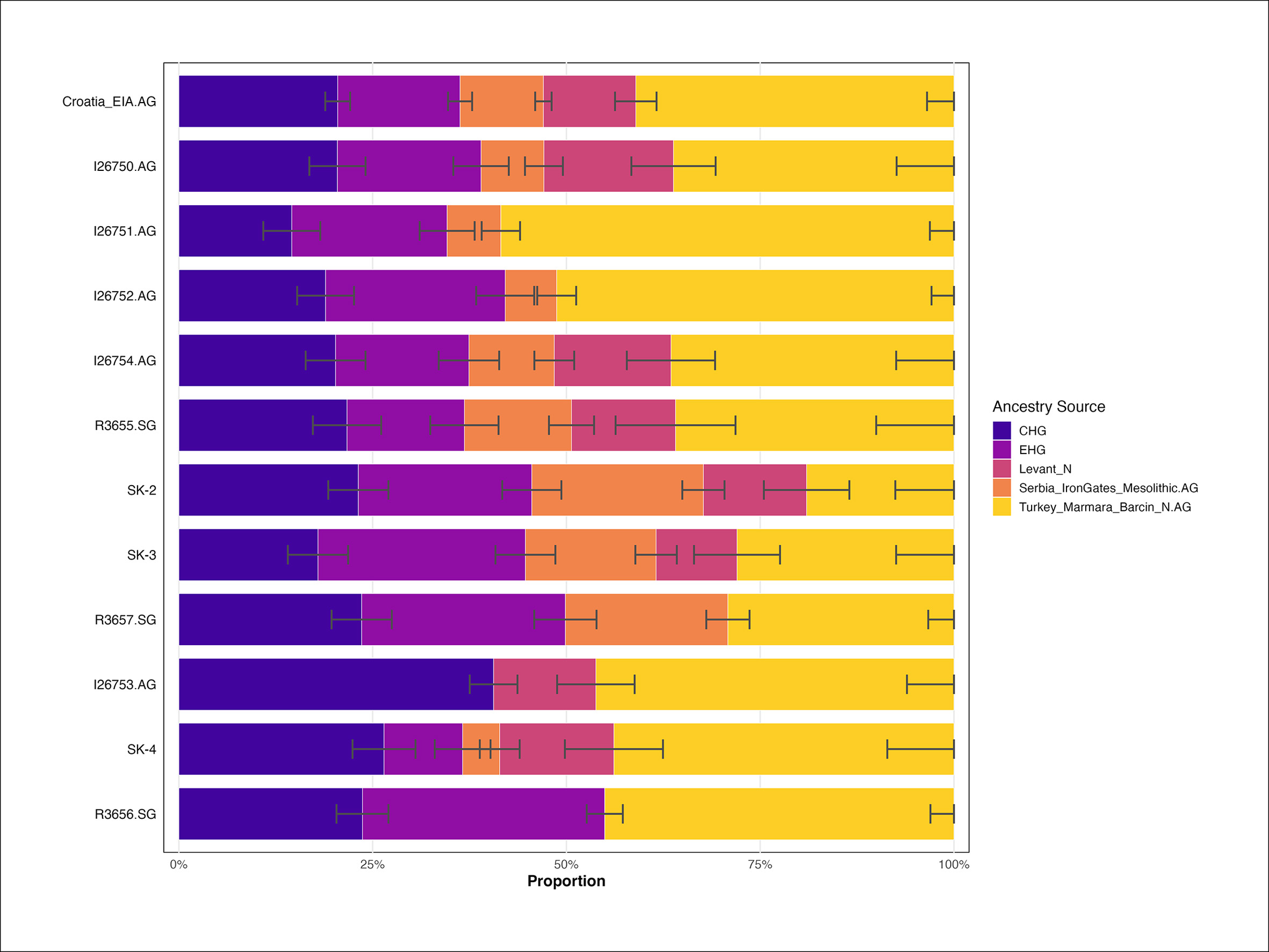

Supplement: S7 Fig — Sample SK 7 could only be modeled with a negative value for one component and is therefore not depicted in this figure (addition information in S1 File: Distal qpAdm). (TIF) [file pone.0333440.s007.tif]
